# Isolated solitary brain metastasis as a relapse of small cell lung cancer

**DOI:** 10.3892/ol.2013.1489

**Published:** 2013-07-24

**Authors:** HIROFUMI SAKURAI, KOICHI KURISHIMA, SHINSUKE HOMMA, KATSUNORI KAGOHASHI, KUNIHIKO MIYAZAKI, MIO KAWAGUCHI, HIROAKI SATOH, NOBUYUKI HIZAWA

**Affiliations:** 1 Division of Respiratory Medicine, Faculty of Medicine, University of Tsukuba, Tsukuba 305-8575, Japan; 2 Division of Respiratory Medicine, Mito Medical Center, University of Tsukuba, Mito, Ibaraki 310-0015, Japan

**Keywords:** isolated solitary brain metastasis, relapse, small cell lung cancer

## Abstract

The brain is one of the most common sites for the metastasis of small cell lung cancer (SCLC). The present study describes two cases of an isolated solitary brain metastasis as a relapse of SCLC, which occurred more than one year after the completion of the initial successful treatment for SCLC. The tumors were identified during a regular check-up computed tomography (CT) scan and were successfully treated. To the best of our knowledge, this is the first study to report the cases of two patients with an isolated solitary brain metastasis as a relapse of SCLC. Although extremely rare, the possibility of such recurrences should be considered, particularly in patients who have refused prophylactic cranial irradiation.

## Introduction

Despite good responses to chemotherapy and chemoradiotherapy, small cell lung cancer (SCLC) is characterized by early and widespread metastases (1). Brain metastases are observed in ~10% of patients at the time of the initial diagnosis, and an additional 40–50% may develop brain metastases during the course of their disease (1). However, late isolated solitary brain metastasis as a relapse of SCLC is rare (2). The present study describes two SCLC patients with an isolated solitary brain metastasis at 18 and 14 months, respectively, following the completion of an initial successful treatment for SCLC. This case report conformed to the Ethical Guidelines for Clinical Studies issued by the Ministry of Health, Labor and Welfare of Japan. Comprehensive informed consent with regard to clinical significance was obtained from the patients.

## Case reports

### Case 1

**Figure 1:**
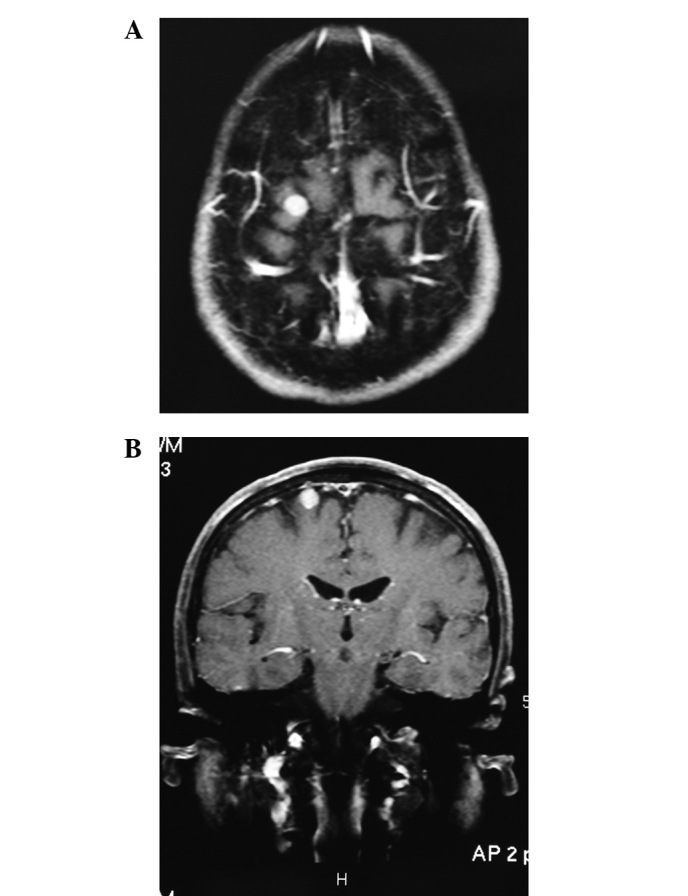
(A and B) Case 1: A metastatic lesion was identified in the right temporal lobe of the cerebral hemisphere during a follow-up magnetic resonance imaging (MRI) examination. (A) Transverse section; (B) coronal section.

A 63-year-old male was admitted to the University of Tsukuba Hospital (Mito, Japan) for an examination of a chest nodule in the right upper lobe of the lung. On admission, the laboratory examination revealed a hemoglobin level of 13.9 g/dl, a hematocrit level of 41.6% and a lactate dehydrogenase level of 139 IU/l. The serum level of neuron-specific enolase (NSE) was 19.9 ng/ml and the pro-gastrin-releasing peptide (proGRP) level was elevated to 522.1 pg/ml. A chest X-ray and computed tomography (CT) scan revealed a poorly-defined mass in the upper lobe of the right lung, with an ipsilateral mediastinal lymph node swelling. A transbronchial biopsy revealed the tumor to be SCLC. As no metastatic lesion was identified, the tumor was diagnosed as limited disease-SCLC. The patient was treated using chemoradiotherapy (chest irradiation up to 65 Gy and four courses of chemotherapy) containing cisplatin (80 mg/m^2^, day 1 for 4 weeks) and etoposide (100 mg/m^2^, days 1–3, for 4 weeks), which resulted in a complete response (CR). A requirement for prophylactic cranial irradiation (PCI) was indicated, however, whole brain irradiation was not administered as the patient did not want the therapy. Subsequent to 18 months from the initial diagnosis of SCLC, a metastatic lesion was observed in the right temporal lobe of the cerebral hemisphere during a follow-up magnetic resonance imaging (MRI) scan (Fig. 1). The patient was administered 30 Gy whole grain irradiation and four courses of platinum-containing chemotherapy consisting of cisplatin (80 mg/m^2^, day 1 for 4 weeks) and etoposide (100 mg/m^2^, days 1–3 for 4 weeks). The patient eventually succumbed to cardiac disease seven years after the recurrence. However, no further recurrence was observed until the patient succumbed.

### Case 2

**Figure 2:**
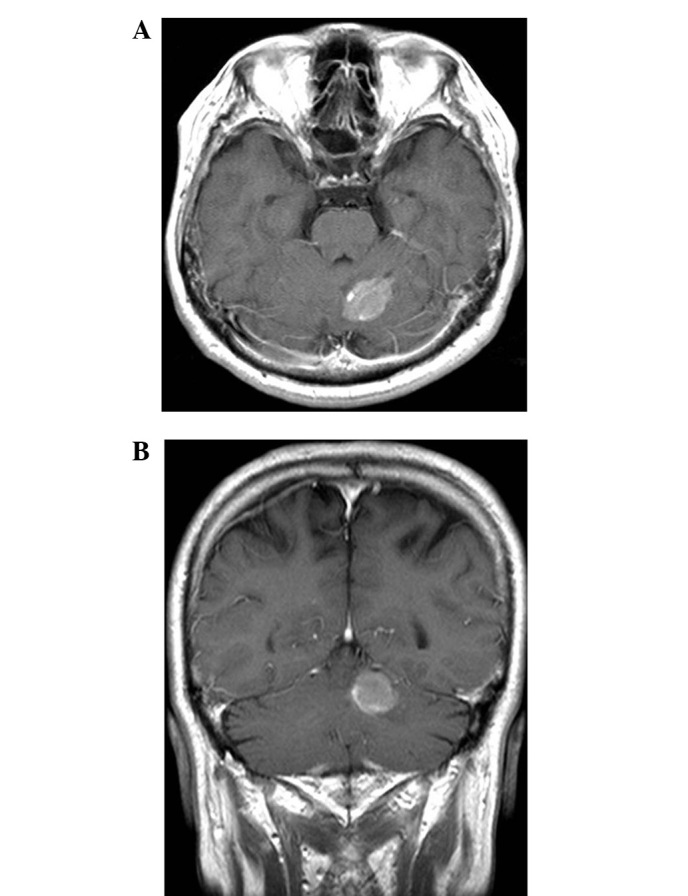
(A and B) Case 2: A metastatic lesion was identified in the left cerebellar hemisphere during a follow-up magnetic resonance imaging (MRI) examination. (A) Transverse section; (B) coronal section.

A 67-year-old male was admitted to Mito Medical Center, University of Tsukuba (Mito, Japan) for an examination of a chest nodule in the right upper lobe of the lung. On admission, the laboratory examination revealed a hemoglobin level of 15.0 g/dl, a hematocrit level of 42.7% and a lactate dehydrogenase level of 223 IU/l. The serum level of NSE was 9.2 ng/ml and the proGRP level was 12.7 pg/ml. A chest X-ray and CT scan revealed a poorly-defined mass in the upper lobe of the right lung, with an ipsilateral mediastinal lymph node swelling. A transbronchial biopsy revealed the tumor to be SCLC. As no metastatic lesion was identified, the tumor was diagnosed as a limited disease-SCLC. The patient was treated using chemoradiotherapy (chest irradiation up to 65 Gy and four courses of chemotherapy) containing cisplatin (80 mg/m^2^, day 1 for 4 weeks) and etoposide (100 mg/m^2^, days 1–3 for 4 weeks), which resulted in a CR. A requirement for prophylactic cranial irradiation (PCI) was indicated, however, it was not administered to the patient as he was concerned about a decline in intellectual level due to the possible neuropsychological problems associated with the treatment. At 14 months after the initial diagnosis of SCLC, a metastatic lesion was identified in the left cerebellar hemisphere on a follow-up MRI scan (Fig. 2). The patient was administered 30 Gy whole grain irradiation and four courses of platinum-containing chemotherapy. The patient remains well at 30 months post-recurrence.

## Discussion

The late relapse of lung cancer has been a growing topic of discussion due to the high level of curability and the possibility of a long survival time in patients. Among the various malignancies, the late relapse of SCLC is well known but uncommon (2–5). In the literature, there have been seven cases of SCLC that have relapsed following ≥10 years of disease-free survival (6–11). In all the cases, the relapse occurred in the intrathoracic region, including in the lungs, the mediastinal lymph nodes or the pleural space. Three cases also experienced brain metastasis (6,8,10). Patients with lung adenocarcinomas rarely develop isolated solitary brain metastasis and certain patients have favorable prognoses (3–5). The present study describes two cases of an isolated solitary brain metastasis as a relapse of SCLC. The literature was searched for the cases of patients with a late isolated solitary brain metastasis as a relapse of SCLC, and only one such case was identified (2). The patient was a long-term survivor of SCLC who was treated with radiation alone and suffered a rare relapse of SCLC, with a solitary brain metastasis 6.5 years after the initial treatment (2). The patient was administered radiation therapy and succumbed to the brain metastasis 8.5 years after the initial treatment (2). An autopsy revealed no tumor recurrence at the primary site and no distant metastases, with the exception of the brain metastasis. The histology of the brain tumor was confirmed to be that of SCLC (2). In the patients of the present study, the brain metastases were identified during a routine follow-up brain CT scan or MRI, without any presenting symptoms. The patients were disease free for more than two years after the successful treatment using whole brain irradiation and additional platinum-containing chemotherapy. A pathological confirmation of the brain metastasis was not obtained. No other disease was located using systemic imaging evaluation at the time of the identification of the brain lesion and there was a good response to the whole brain irradiation and subsequent chemotherapy. The tumors were evaluated clinically as brain metastases from SCLC. With the exception of patients with tumors of the lung, there have only been two patients with isolated brain metastasis as the sole manifestation of a late relapse (12,13). One case was of a late onset of isolated intracranial metastasis of a liposarcoma in the right lower extremity (12). The relapse was identified 26 years after the initial therapy (12). The other case was of an isolated brain metastasis in a patient with breast cancer nine years after the initial therapy (13). In the two cases, additional therapy was unsuccessful in controlling the disease progression (12,13). The mechanism of this form of rare metastasis remains to be elucidated. SCLC cells metastasize at a certain point of their clinical course and may survive at the metastatic sites, escaping from the immune mechanism with no rapid growth. Following a period of long-term dormancy, the cells may then initiate growth again (14).

PCI has been proposed as a form of treatment in SCLC that reduces the incidence of brain metastases and significantly improves overall survival in limited- and extensive disease-SCLC in patients who respond to first-line treatment (15). It has been suggested that this treatment may increase neuropsychological syndromes and brain abnormalities, as indicated by CT scans (16,17). However, no significant increase in late sequelae has been shown in clinical trials between patients with and without PCI (14,18,19). As the patients in the present study had the limited disease form, they were informed that there were indications that PCI should be performed at the time of their good response to first-time treatment. The patients refused PCI for fear of the appearance of neuropsychological symptoms. However, once diagnosed with an isolated solitary brain metastasis, the patients underwent whole brain irradiation. The two patients developed no neuropsychological symptoms.

To the best of our knowledge, the two cases of isolated solitary brain metastasis relapse of SCLC of the present study are the first to have been described. As shown in the present study, there are patients who may be expected to have long-term survival following the additional therapy for brain metastasis, therefore careful follow-up is necessary to detect metastatic lesions as early as possible, particularly for patients who have refused PCI.
